# Impact of Multistrain Probiotic Supplementation on Glycemic Control in Type 2 Diabetes Mellitus—Randomized Controlled Trial

**DOI:** 10.3390/life14111484

**Published:** 2024-11-14

**Authors:** Venkata Chaithanya, Janardanan Kumar, Kakithakara Vajravelu Leela, Mohan Ram, Jayaprakash Thulukanam

**Affiliations:** 1 Department of Microbiology, SRM Medical College Hospital and Research Centre, SRMIST, Kattankulathur, Chengalapattu 603203, Tamil Nadu, India; vd6943@srmist.edu.in (V.C.); leelav@srmist.edu.in (K.V.L.); jpmicro74@gmail.com (J.T.); 2 Department of General Medicine, SRM Medical College Hospital and Research Centre, SRMIST, Kattankulathur, Chengalapattu 603203, Tamil Nadu, India; 3 Department of Medical Laboratory Technology, SRM Medical College Hospital and Research Centre, SRMIST, Kattankulathur, Chengalapattu 603203, Tamil Nadu, India; mm0244@srmist.edu.in

**Keywords:** type 2 diabetes, probiotics, glycemic control, HbA1c, metabolic health

## Abstract

Hyperglycemia, a key characteristic of type 2 diabetes mellitus (T2DM), highlights the need for effective management strategies. This study aims to analyze the impact of multistrain probiotic supplementation on glycemic control in T2DM patients. During a 24-week randomized controlled trial involving 130 participants, subjects were assigned to either a probiotic group or a placebo group. The key outcomes included fasting blood glucose (FBG), postprandial blood glucose (PPBG), glycated hemoglobin (HbA1c) levels, and lipid profiles, assessed at baseline and post-intervention. The results indicated a significant reduction in HbA1c (*p* = 0.004) and increased HDL-c (*p* = 0.023) and improvements in lipid profiles in the probiotic group, alongside a trend toward decreased FBG and PPBG. No serious adverse effects were reported, indicating good tolerance of probiotics. These findings suggest that probiotics may positively influence metabolic parameters in T2DM patients, supporting their potential as a complementary dietary intervention. Further research is needed to understand the underlying mechanisms and enhance probiotic formulations for diabetic control.

## 1. Introduction

In an era in which lifestyle diseases are on the rise, type 2 diabetes mellitus (T2DM) stands out as a formidable public health challenge, affecting millions globally [1]. As the incidence of this metabolic disorder continues to grow, the need for effective management strategies becomes increasingly critical. Among these strategies, dietary interventions, particularly those focusing on glycemic control, have emerged as vital components in controlling blood sugar levels and preventing complications [2].

Probiotics, constituted by live microorganisms that offer health benefits when consumed in suitable proportions, have recently attracted considerable interest regarding their potential impact on metabolic health, particularly glycemic control [3]. The human gut microbiota performs a significant role in glucose metabolism, and disruptions in its balance, referred to as dysbiosis, have been related to the development and progress of T2DM [4]. Research indicates that probiotic supplementation can positively alter gut microbiota composition, which may subsequently affect glucose metabolism and enhance insulin sensitivity [5,6,7].

Studies have examined the influence of probiotics on several metabolic health markers in T2DM patients. Many of these investigations have reported notable improvements in glycemic control indicators, such as reductions in fasting blood glucose (FBG) and lower glycated hemoglobin (HbA1c) levels. The potential mechanisms by which probiotics influence glycemic control are multifaceted, including modulation of gut hormone secretion, improvement of intestinal barrier function, and reduction in systemic inflammation [8,9].

Understanding the relationship between probiotics and glycemic control is essential, as enhancing glycemic responses through probiotic supplementation could provide an innovative dietary strategy for managing blood glucose levels in T2DM patients. 

The present study aims to investigate the effects of probiotic supplementation on glycemic control among individuals with T2DM. By assessing changes in glycemic responses following the introduction of probiotics along with antidiabetic drugs, we aim to shed light on the potential benefits that probiotics may offer in modulating glycemic control. The implications of this study extend beyond just the immediate effects on glycemic control. If probiotics are shown to effectively lower blood sugar levels, this could lead to more tailor-made recommendations for individuals with T2DM [10,11].

Moreover, the potential synergistic effects between probiotics and conventional antidiabetic medications could open new avenues for combination therapies that enhance overall treatment efficacy [12]. By investigating this underexplored area, we hope to pave the way for innovative strategies that can assist in the effective management of this prevalent metabolic disorder.

## 2. Materials and Methods

### 2.1. Study Design

This 24-week randomized controlled trial was carried out at the diabetic clinic of SRM Medical College Hospital and Research Centre. The research protocol was approved by the institutional ethical committees (Ethics Clearance Number: 8519/IEC/2023; 30 April 2023) at the SRM Medical College Hospital and Research Centre and registered with the Clinical Trials Registry—India under the trial number CTRI/2023/07/055647.

### 2.2. Selection of Participants

Participants were screened for the study between August 2023 and October 2023 at the diabetes clinic. A total of 130 individuals with (T2DM), comprising 71 females and 51 males, were recruited. These participants were randomly assigned to one of two groups: those receiving multispecies probiotic supplements (*n* = 65, made up of 22 males and 43 females) or those receiving a placebo (without probiotics) (*n* = 65, made up of 29 males and 36 females). The study’s approach was thoroughly explained to all participants, who signed informed consent forms before enrolling.

The eligibility criteria for this study were as follows: participants had to have an established diagnosis of T2DM for at least 6 months prior to the beginning of the study, must not be currently on antibiotics, must be aged between 25 and 70 years, and had to have a glycated hemoglobin A1c (HbA1c) level of 6.5 or higher. Exclusion criteria included individuals with type 1 diabetes, pregnant women, those with gestational diabetes, and participants who had already begun taking probiotic supplements.

Participants in the probiotic group were instructed to take the supplement capsules twice daily, once in the morning and once at night, before meals. They were directed to maintain their usual physical activity levels and nutritional habits during the trial. Furthermore, participants were instructed not to take any additional probiotic supplements during the trial. To monitor compliance, capsule consumption was assessed through weekly mobile interviews.

### 2.3. Characteristics of Supplements

The multispecies probiotic supplement utilized in this study contained a combination of fourteen live strains of microorganisms, with a total of 30 billion colony-forming units (CFUs) per dose. The strains included in the supplement were *Lactobacillus plantarum* (*L. plantarum*), *L. fermentum*, *L. acidophilus*, *L. casei*, *L. rhamnosus*, *L. reuteri*, *L. salivarius*, *L. paracasei*, *L. gasseri*, *Bifidobacterium bifidum* (*B. bifidum*), *B. lactis*, *B. breve*, *Streptococcus thermophilus*, and *Saccharomyces boulardii.* Participants were instructed to store the capsules away from direct sunlight to preserve viability.

### 2.4. Measurement of Outcomes

The primary objective measured was glycemic control. Secondary outcomes included diabetes-related factors like body mass index (BMI), lipid profile, and blood pressure. Blood samples were obtained following a 10 to 12 h fast. FBG and 2 h postprandial blood glucose (PPBG) levels were determined utilizing enzymatic techniques. Enzymatic assays were used to assess the lipid profile, which included measurements of total cholesterol (TC) and triglycerides (TGL). The direct method was used to quantify low-density lipoprotein cholesterol (LDL-c). The levels of very-low-density lipoprotein cholesterol (VLDL-c) were determined using TGL. HDL-c levels were measured utilizing a direct antibody inhibition technique. FBG, PPBG, and lipid profiles were measured by Beckman coulter DxC 700 AU. HbA1c levels were determined using high-performance liquid chromatography (HPLC) by D-10, Bio-Rad, a dependable method for assessing long-term glycemic management. All the enzymatic kits used for analysis were sourced from Beckman coulter. BMI assessment was calculated using the following formula: weight (kg)/height (m^2^). The participants’ weight and height were measured using standardized equipment to ensure accuracy. Blood pressure was assessed using a calibrated sphygmomanometer. Measurements were taken in a seated position after a five-minute rest, with an average of two readings recorded to ensure reliability. Nutritional habits and nutritional intake were assessed using a 24 h dietary recall, which provided a comprehensive analysis of the participants’ nutritional intake. The dietary data were analyzed by a dietician in a diabetic clinic.

### 2.5. Statistical Analysis

Analysis of statistics was performed with SPSS software, version 25. A significance level of *p* < 0.05 was established to identify significant differences between and within the groups. To analyze differences within groups before and after the intervention, paired sample *t*-tests were utilized. Independent Student *t*-tests were utilized to identify changes among the probiotic and placebo groups, allowing for a comprehensive assessment of the efficacy of the probiotic intervention.

## 3. Results

**Figure 1:**
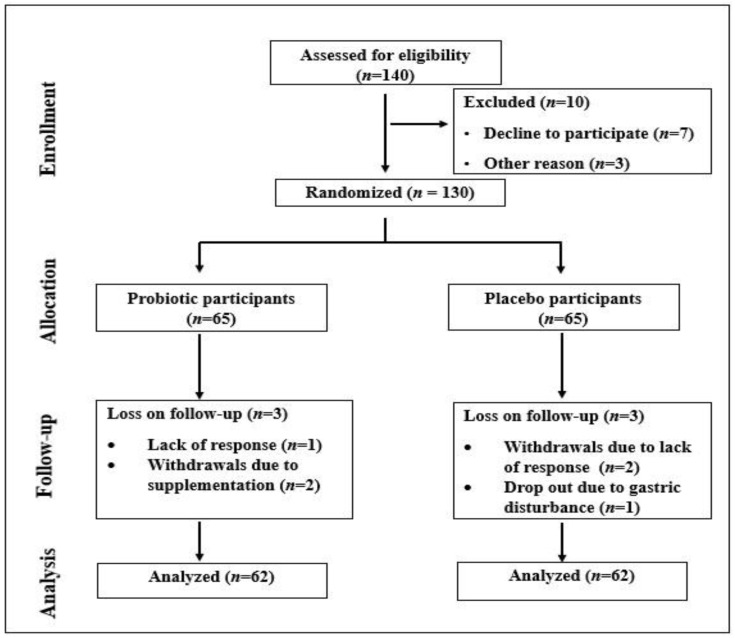
CONSORT flowchart.

A total of 124 people completed the trial in this study, with 62 individuals in the probiotic group and 62 in the placebo group. During the trial, three patients from the multispecies probiotic group were excluded: one due to a lack of response and two who withdrew because of supplementation. Similarly, three participants in the placebo group were excluded—two for a lack of response and one for gastric disturbances (Figure 1). Importantly, no serious adverse reactions were reported among participants who consumed the multispecies probiotic supplements throughout the study, indicating that the probiotics were well tolerated by patients with T2DM.

The demographic data, including average height and age, were similar across the two groups, allowing for any observed differences in outcomes to be attributed to the probiotic intervention rather than variations in participant characteristics. At baseline, there were no statistically significant variations in weight and BMI between the two groups (Table 1). This suggests that the groups started from a similar physiological state and remained comparable throughout the study. Regarding dietary intake, participants in both groups had similar nutritional habits at the beginning of the study. Analyzing the nutritional intakes during the run-in period and throughout the study, we found no changes in within-group differences for dietary patterns, reinforcing the notion that the results were not influenced by changes in diet. This careful monitoring of dietary intake helps strengthen the validity of the findings, as it minimizes confounding factors related to nutrition.

**Figure 2:**
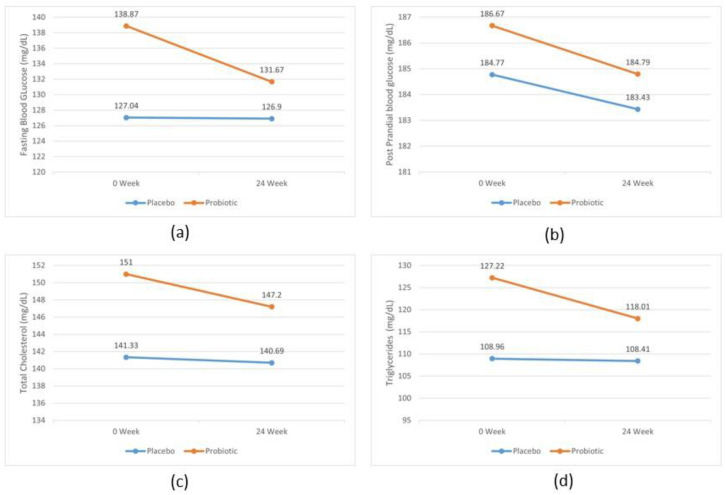

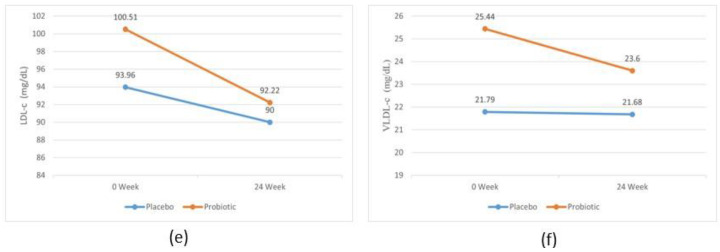
Analysis of (**a**) fasting blood glucose, (**b**) postprandial blood glucose, (**c**) total cholesterol, (**d**) triglycerides, (**e**) low-density lipoprotein cholesterol, and (**f**) very-low-density lipoprotein of placebo and probiotic group at 0th and 24th week. Data expressed in means.

At baseline, no significant variations in biochemical measures were observed between the probiotic and placebo group, which sets a solid foundation for evaluating the effects of the probiotics. However, supplementation with a live multistrain of probiotics for 24 weeks significantly reduced BMI in the probiotic group, decreasing from 27.3 ± 4.13 to 26.7 ± 4.04 (*p* < 0.001). In comparison, the placebo group failed to observe any significant change in BMI. While the probiotic group showed significant outcomes, there were no remarkable differences in BMI between the placebo and probiotic groups, indicating that although probiotics had a positive effect on reducing BMI, it did not translate to a significant advantage over the placebo in this aspect. Blood pressure measurements showed no statistically significant differences within or between the placebo and probiotic groups, suggesting that the probiotic supplementation did not impact blood pressure levels. Similarly, FBG and 2 h PPBG levels exhibited no significant changes between the two groups, although the mean difference (MD) was noticeable in the probiotic group compared to the placebo (MD-FBG −7.19, PPBG −1.88 probiotic vs. −0.1, −1.33 placebo) (Figure 2). This indicates a trend toward improved glycemic control in the probiotic group, even if it failed to attain statistical significance. When examining HbA1c levels, we found a significant reduction in the probiotic group (*p* = 0.004), highlighting the potential of probiotics to improve long-term glycemic control. Moreover, a significant difference was observed when comparing changes in HbA1c between placebo and probiotic groups (*p* = 0.041) (Table 2), suggesting that the probiotic intervention had a meaningful impact on this important metabolic marker.

**Figure 3:**
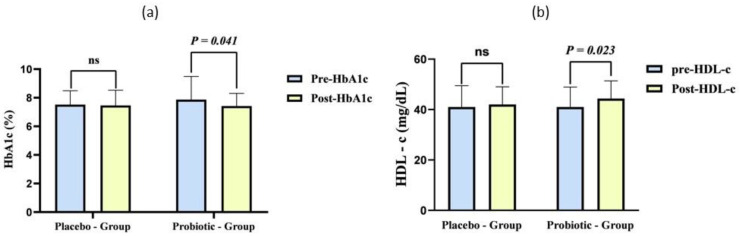
Analysis of (**a**) Hba1c and (**b**) HDL-c at baseline (pre) and at 24th week (post).

Regarding lipid profiles, the probiotic group exhibited larger decreases in total cholesterol (−3.83 ± 34.67 vs. −0.64 ± 24.09 mg/dL in placebo, *p* = 0.556), triglycerides (−9.20 ± 47.53 vs. −0.54 ± 30.44 mg/dL in placebo, *p* = 0.233), and LDL cholesterol (−8.29 ± 30.65 mg/dL vs. −3.96 ± 19.74 mg/dL in placebo, *p* = 0.356). The probiotic group exhibited more substantial changes. HDL cholesterol, often referred to as “good” cholesterol, increased significantly in the probiotic group (3.33 ± 4.87 mg/dL, *p* < 0.001) compared to a smaller improvement in the placebo group (1.45 ± 4.12 mg/dL, *p* = 0.008). The between-group difference in HDL cholesterol change was statistically significant (*p* = 0.023) (Figure 3), indicating a positive effect of probiotics on HDL levels. VLDL cholesterol showed a larger decrease in the probiotic group (−1.84 ± 9.50 mg/dL) compared to the placebo (−0.10 ± 6.08 mg/dL), but this variation failed to show statistical significance (*p* = 0.233). It is crucial to consider that where numerous measures exhibited enhancements in the probiotic group, these did not attain statistical significance in intergroup comparisons.

A notable decrease in LDL-c values was recorded in the probiotic group (*p* = 0.039). Nonetheless, we observed no significant difference in LDL-c alterations between the placebo and probiotic groups, suggesting that although probiotics may aid in reducing LDL-C, this impact did not markedly differ from that observed in the placebo group.

## 4. Discussion

Recent research has highlighted the importance of gut microbiota in the etiology of T2DM. Probiotics have been recommended as an effective method of altering gut microbiota and may provide specific health benefits. However, the impact of probiotic supplementation on metabolic profiles in T2DM remains a topic of ongoing debate. Dysbiosis in gut microbiota can influence inflammatory pathways and disrupt energy metabolism, potentially affecting glucose and lipid metabolism as well as insulin sensitivity. Emerging research suggests that probiotics may help modify gut flora, leading to improved cholesterol levels and reduced blood glucose [13,14]. A meta-analysis involving twelve RCTs indicated that probiotics could lower FBG levels by approximately 15 mg/dL and HbA1c by 0.54% [15]. In contrast, our study observed reductions of about 7 mg/dL in FBG and 0.44% in HbA1c, suggesting that multispecies probiotics can prevent elevations in FBG, indicating a modest effect on glycemic control. Our study also a observed minimal reduction in 2 h PPBG when compared with the placebo. The mechanism of decreasing blood glucose is still not fully understood. This is because probiotic supplementation largely lowers FBG through better insulin sensitivity, decreased inflammation, and altered gut microbiota composition, whereas its effect on 2 h PPBG is less significant due to the acute nature of meal-induced glucose response. The complicated interaction of these factors limits their influence on postprandial glucose control. They may also be associated with lower oxidative stress [16], which has been demonstrated in hyperglycemia. Specific strains of lactic acid bacteria have antioxidant properties. Yadav et al. [17] found that *Lactobacillus acidophilus* and *L. casei* delayed the progression of hyperglycemia and hyperinsulinemia in animal models by reducing oxidative stress. Probiotics may attenuate systemic endotoxin, thereby influencing glucose metabolism [18].

Many individuals with diabetes experience dyslipidemia, which can lead to oxidative stress and increase the risk of complications such as cardiovascular disease, diabetic nephropathy, retinopathy, and hypertension [19,20]. We observed a notable decrease in total cholesterol and LDL-c in both groups, with a greater reduction among those receiving probiotics. The mechanisms of reducing lipid metabolism include modulation of gut microbiota, which enhances the production of short-chain fatty acids (SCFAs) and regulates lipolysis. These SCFAs inhibit endogenous lipolysis and regulate extracellular lipolysis mediated by an increase in lipoprotein lipase expression, resulting in a decrease in the circulating lipid plasma levels [21,22].

A meta-analysis by Hu et al. [23] involving 770 participants also reported that probiotics significantly reduced TC and TGL while increasing HDL-c. However, our study found no significant changes in TC, TGL, and VLDL-c both within and between the groups. The study observed a significant decrease in LDL-c (*p* = 0.039) and an increase in HDL-c (*p* < 0.001) in the probiotic group, with significant variations within this group. Previous clinical trials [24,25,26,27,28,29,30] have produced mixed results regarding the effects of probiotics on glycemic control. Some studies have reported improvements after probiotic supplementation or consumption of fermented milk products [24,26,27,30], while others have not observed any beneficial effects in T2DM [25,29] and obese individuals [31,32]. The current study found a significant reduction in HbA1c and an increase in HDL-c. A meta-analysis by Kasinska et al. [7] involving eight trials and 438 participants also noted a significant effect of probiotics on HbA1c levels.

Moreover, the composition of the gut microbiota can be influenced by dietary patterns, which means that a holistic approach that includes dietary interventions alongside probiotic supplementation could yield better outcomes. For instance, diets rich in fiber, such as those high in fruits, vegetables, and whole grains not only support the growth of beneficial bacteria but also synergize with probiotics to enhance their effectiveness [33]. Despite the promising findings regarding probiotics, challenges remain in standardizing probiotic formulations and identifying the most effective strains for specific metabolic conditions. Individual responses to probiotics can vary significantly based on genetic, environmental, and lifestyle factors, making it difficult to generalize results across different populations [34]. Our study population’s dietary patterns typically included a high consumption of rice, legumes, vegetables, and traditional spices. However, urban areas are seeing a growing trend toward refined carbohydrates, such as white rice, processed snacks, and sugars, especially among younger individuals [35]. Research shows that diets high in refined carbs and sugars adversely affect glucose metabolism and gut microbiota composition [36]. Compared to fiber-rich, plant-based diets, such as those in Mediterranean regions, our study population may result in different metabolic responses. These dietary and microbiota differences may limit the generalizability of our findings to other populations with different dietary patterns.

Our findings suggest that probiotics potentially lead to favorable metabolic changes with consistent use. The administration of probiotics daily for 24 weeks demonstrated improvements in FBG, PPBG, HbA1c, and lipid profiles in T2DM patients. The impact of multistrain probiotic supplementation on glycemic control in type 2 diabetes mellitus (T2DM) presents strengths and limitations. The strength of the study is that the use of a multistrain approach enhances the potential therapeutic effects by targeting different aspects of glycemic control, such as improving HbA1c and decreasing FBG. These factors lend this study high clinical relevance, potentially providing an adjunctive therapy for T2DM. However, the limitation of the study is its small sample size. Furthermore, probiotic supplements are not standardized; thus, their efficiency varies between strains. So, the findings of the present study may not apply to all probiotics currently on the market. Future research with larger and more diverse populations is needed to solidify the clinical utility of probiotics for glycemic control in T2DM.

## 5. Conclusions

As the understanding of the gut microbiome continues to evolve, integrating probiotics into comprehensive diabetes management plans may offer new avenues for improving metabolic health and reducing the burden of T2DM. This 24-week study demonstrates the promising effects of probiotic supplementation on HbA1c and HDL cholesterol levels, with positive trends in other metabolic parameters. The lack of statistical significance in many parameters, despite observable trends toward improvement, indicates that larger, longer-term studies may be needed to fully elucidate the effects of probiotic supplementation on metabolic health and lay the groundwork for more extensive clinical investigations in this field. Continued research will be critical in harnessing the full potential of probiotics as a therapeutic strategy in the fight against diabetes and its associated complications.

## Figures and Tables

**Table 1:** Baseline characteristics of study participants.

	Placebo	Probiotic	*p* ^a^
Age	57.6 ± 8.56	55.0 ± 9.16	0.111
Height, cm	157.3 ± 8.08	157.2 ± 8.05	0.930
Weight—baseline, kg	70.8 ± 11.67	67.6 ± 10.9	0.092
BMI—baseline	28.6 ± 5.04	27.3 ± 4.13	0.127
Systole—baseline, mmHg	130.2 ± 19.03	127.3 ± 17.45	0.390
Diastole, mmHg	79.5 ± 9.96	79.7 ± 9.29	0.993
FBG, mg/dL	127.0 ± 27.66	138.8 ± 44.81	0.082
2 h PPBG, mg/dL	184.7 ± 52.27	186.6 ± 60.97	0.852
HbA1c, %	7.51 ± 0.96	7.86 ± 1.61	0.147
Total cholesterol, mg/dL	141.2 ± 33.80	151.0 ± 42.32	0.158
Triglycerides, mg/dL	108.9 ± 44.07	127.2 ± 70.26	0.088
LDL-c, mg/dL	93.9 ± 28.46	100.5 ± 33.79	0.249
HDL-c, mg/dL	41.0 ± 8.45	41.0 ± 7.88	0.991
VLDL-c, mg/dL	21.7 ± 8.81	25.4 ± 14.05	0.088

Data are represented as mean ± SD. ^a^ Obtained from an independent Student *t*-test for the between-group comparisons. cm = centimeter; kg = kilogram; mmHg = millimeters of mercury, FBG = fasting blood glucose; PPBG = postprandial blood glucose; HbA1c = glycated hemoglobin; LDL-c = low-density lipoprotein cholesterol; HDL-c = high-density lipoprotein cholesterol; VLDL-c = very-low-density lipoprotein cholesterol; mg/dL = milligrams per deciliter.

**Table 2:** Within-group and between-group comparisons of metabolic profiles.

	Placebo				Probiotic Supplementation				
	Week 0	Week 24	Change	*p* ^a^	Week 0	Week 24	Change	*p* ^a^	*p* ^b^
FBG, mg/dL	127.0 ± 27.6	126.9 ± 31.3	−0.1 ± 28.7	0.969	138.87 ± 44.81	131.67 ± 28.77	−7.19 ± 41.97	0.186	0.281
2 h PPBG, mg/dL	184.7 ± 52.2	183.4 ± 64.4	−1.3 ± 66.9	0.876	186.67 ± 60.97	184.79 ± 52.26	−1.88 ± 65.49	0.823	0.964
HbA1c, %	7.51 ± 0.9	7.46 ± 0.96	−0.05 ± 0.92	0.666	7.86 ± 1.61	7.42 ± 0.88	−0.44 ± 1.17	0.004 *	0.041 *
Total Cholesterol, mg/dL	141.33 ± 33.80	140.69 ± 26.24	−0.64 ± 24.09	0.835	151.09 ± 42.32	147.25 ± 37.54	−3.83 ± 34.67	0.391	0.556
Triglycerides, mg/dL	108.96 ± 44.07	108.41 ± 37.98	−0.54 ± 30.44	0.889	127.22 ± 70.26	118.01 ± 47.30	−9.20 ± 47.53	0.135	0.233
LDL-c, mg/dL	93.96 ± 28.4	90.0 ± 23.69	−3.96 ± 19.74	0.122	100.51 ± 33.74	92.22 ± 29.71	−8.29 ± 30.65	0.039 *	0.356
HDL-c, mg/dL	41.03 ± 8.45	42.48 ± 7.81	1.45 ± 4.12	0.008 *	41.04 ± 7.88	44.38 ± 7.42	3.33 ± 4.87	<0.001 *	0.023 *
VLDL-c, mg/dL	21.79 ± 8.81	21.68 ± 7.59	−0.10 ± 6.08	0.889	25.44 ± 14.05	23.60 ± 9.46	−1.84 ± 9.50	0.135	0.233

Data are represented as mean ± SD. ^a^ Obtained from a paired *t*-test for the within-group comparisons. ^b^ Obtained from an independent Student *t*-test for the between-group comparisons. FBG = fasting blood glucose; PPBG = postprandial blood glucose; HbA1c = glycated hemoglobin; LDL-c = low-density lipoprotein cholesterol; HDL-c = high-density lipoprotein cholesterol; VLDL-c = very-low-density lipoprotein cholesterol; mg/dL = milligrams per deciliter. Statistically significant values are denoted as “*”.

## Data Availability

The data presented in this study are available on request from the corresponding author due to ethical reasons.
